# Parental perspectives on the changes in their child’s participation in physical activities after a highly intensive functional balance training for Developmental coordination disorder: A sequential multimethod qualitative study

**DOI:** 10.1371/journal.pone.0331994

**Published:** 2026-05-14

**Authors:** Silke Velghe, Ingrid Van der Veer, Marieke Coussens, Eugene Rameckers, Pieter Meyns, Barbara Piskur, Evi Verbecque, Katrijn Klingels

**Affiliations:** 1 Rehabilitation Research Centre - REVAL, Faculty of Rehabilitation Sciences, Hasselt University, Diepenbeek, Belgium; 2 Centre of Expertise, Adelante Rehabilitation Centre, Valkenburg, the Netherlands; 3 Faculty of Medicine and Health Sciences, Department of Rehabilitation Science, Ghent University, Ghent, Belgium; 4 Department of Rehabilitation Medicine, Functioning, Participation; Rehabilitation research line, Research School CAPHRI, Maastricht University, Maastricht, the Netherlands; 5 Research Center Autonomy and Participation, Zuyd University of Applied Science, Heerlen, the Netherlands; Prince Sattam bin Abdulaziz University, SAUDI ARABIA

## Abstract

**Background:**

Children with Developmental coordination disorder (DCD) experience challenges that extend beyond the motor domain, impacting their mental health and participation but also their parents and families. Currently, little is known about how motor intervention affects participation in physical activities in children with DCD.

**Methods:**

This sequential multimethod qualitative study explored parental perspectives on their child’s participation following a highly intensive one-week (40-hour) group intervention focusing on balance performance in school-aged children with DCD. In Phase 1, post-intervention changes in participation in physical activities were examined using open-ended questionnaires in a first cohort of parents. In Phase 2, focus groups with a second cohort of parents further explored the changes of Phase 1 and investigated how these were interrelated. Thematic analysis was applied in both phases.

**Results:**

Parents (Phase 1: 22; Phase 2: 14) reported improvements in their child’s participation, family dynamics and social interactions. Key themes included increased child empowerment, increased participation involvement through self-initiated actions, parent-centered support, and the fact that the empowerment of both parents and child potentially induced sustained changes in their child’s participation in physical activities after three months. Specific interventional features (individual therapist, accessible environment, and high amount of therapy time) empowered the child, initiating greater participation involvement through self-initiated actions. These changes were sustained through enhanced parental understanding of DCD and improved communication with their child and members of their environment (e.g., teachers), leading to shifts in parental behavior and sustained changes in daily routines.

**Conclusion:**

This highly intensive group-based functional balance training resulted in sustained improvements in child participation in physical activities after three months potentially due to empowering both the child and their parents. These findings highlight the importance of addressing not only motor performances of the child with DCD but also involve caregivers in therapeutic processes to attain sustained changes.

## Introduction

Developmental coordination disorder (DCD) affects 5–6% of school-aged children and is characterized by clumsy motor behavior [1]. These motor problems affect skills needed to participate in daily life [2–4], frequently resulting in social exclusion [5] and avoidance or withdrawal from participation [3,6]. The International Classification of Functioning (ICF) defines participation as ‘involvement in a life situation’ [7], however, literature highlights its complex and multi-dynamic nature [8–10]. Imms and colleagues developed the family of Participation Related Constructs (fPRC) model as a theoretical framework to clarify the concept of participation and support participation-based research and practice [11]. The fPRC model addresses issues at the level of the individual in a participation context within an overarching environmental framework and describes processes within and between the different elements of the model [11]. It defines participation as a combination of attendance (‘being there’) and involvement (‘the quality of the experience when being there’) [11]. The participation difficulties in children with DCD comprise both attendance and involvement and are associated with psychosocial problems, with higher rates of depression and anxiety when compared to typically developing peers [3,12–14]. Having DCD not only impacts the child, but also their parents and family. For instance, parents report problems with their child in areas such as dressing, hygiene, organization, and homework [4,15] requiring extra parental time to support the child during daily life activities and limiting the amount of available personal time [16,17]. This negatively impacts the parental work-life balance [18] resulting in feelings of fatigue, guilt and emotional distress [17]. In families with more than one child, siblings reported receiving less parental time compared to their sibling with DCD, which can cause jealousy, but also feelings of embarrassment and frustration [18]. Additionally, at the family level, the child’s movement difficulties limit the type of activities that can be done together as a family [16–18].

Next to the above-mentioned practical and emotional challenges, parents of children with DCD experience stress, worries, and concerns about their child’s future, withdrawal from and learning of new physical activities [16,17,19,20]. The motor difficulties experienced by children with DCD are not only correlated with these parental concerns but may also serve as an independent predictor of parental stress [19,21]. Thus, addressing these motor difficulties through targeted interventions could potentially alter these parental concerns. Hung et al. showed that parents of children with DCD perceived a functional motor skill intervention as beneficial for their children and themselves, and can alter parental behavior [22]. After the intervention, they felt more psychologically supported and motivated to play an active role in physiotherapy management [22]. More specifically, following highly intensive therapy, parents reported higher satisfaction and performance rates of the personal goals of their child [23,24], increased understanding of the disorder [23], improvements in the child’s attitude, motivation [24] and greater confidence related to performing motor activities [23,24].

One of the motor difficulties in children with DCD is impaired postural control, which is highly prevalent and heterogeneous [25,26]. Yet, current therapy approaches do not address all postural control systems and fail to align with international DCD guidelines [27], which emphasize goal-oriented, task- and context-specific therapy at the activity and participation levels of the ICF [1,7]. To bridge this gap, a comprehensive group-based functional balance training was developed based on three principles. First, the intervention comprises all postural control systems included in functional activities such as balance bikes, obstacle relay with dual tasks and circus barrel walking. Second, it aligns with the international DCD guidelines by being goal-oriented, task-specific, and context-based. Third, highly intensive therapy (defined as at least 30 therapy hours with a minimum frequency of three times per week [28] has proven effective in children with Cerebral palsy (CP) for improving mobility, self-care, and physical well-being [29–31]. While less commonly applied in DCD, initial findings also suggest benefits of highly intensive therapy for individual goal achievement, functional gait, and gait stability [23,24,32,33]. However, to our knowledge, its effects on postural control remained unexplored. To address this, a comprehensive functional group-based training was designed to intensively target balance in children with DCD [34].

Since children with DCD show significantly reduced participation involvement, particularly in school and community settings, compared to their typically developing peers [3,35], the current qualitative study specifically examines effects at the participation level. A systematic review shows that individually tailored interventions can improve children’s participation [36]. However, in children with DCD, only two studies have examined the effects of such interventions on participation [23,24]. Both studies applied the Cognitive Orientation to Daily Occupational Performance (CO-OP) approach and primarily reported quantitative participation outcomes [23,24]. One study also included parent interviews but found limited effects on participation [23,24]. It remains unclear how balance-related individually tailored training affects a child’s participation. Since balance activities are key in daily life of school-aged children [12,25,37], findings on participation outcomes can be different from previous studies.

As such, this qualitative study aims to provide insights in the perceived effects of a highly intensive group-based functional balance training on the participation in physical activities of school-aged children with DCD. As a first step, we explored parents’ perspectives about their perceived changes on their child’s participation in physical activities following the intervention (Phase 1). Based on these findings, focus groups were organized in a new cohort (Phase 2) to explore the perceived changes found in Phase 1 in more detail and investigate how these changes are interrelated.

## Materials and methods

### Study design and research question

This exploratory, sequential multimethod qualitative study [38,39] consisted of two Phases. In Phase 1, open-ended questionnaires were conducted with parents to explore their perspectives on their perceived changes on their child’s participation in physical activities following the intervention. In Phase 2, focus groups were performed to (a) gain deeper insights in the perceived changes of Phase 1 and (b) to explore how these changes are interrelated.

**Fig 1:**
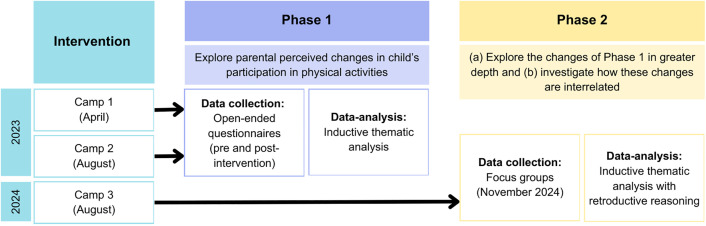
Overview of qualitative study-design. NOTE. Interventions in Camps 1, 2 and 3 are identical. Arrows indicate the population, identified as parents of participants.

Since the data analysis of Phase 1 needed to be completed to progress to Phase 2, there was a long time interval between the end of Phase 1 (Camp 1 and 2) and the start of Phase 2. As Phase 2 involved focus groups, in which parents discussed specific behaviors, observations, and feelings during and after the intervention period, the time gap could have resulted in recall bias if it had been conducted with the same participants of Phase 1, as they might remember or interpret past experiences differently over time. Therefore, Phase 2 was conducted in a new cohort of parents (Camp 3) (Fig 1). Participants of the intervention were recruited from 15 September 2022 till 28 March 2024. Invitation of the parents to join the qualitative part of the study took part after inclusion of their child(ren) at specific timepoints relative to the intervention (Phase 1: before the intervention; Phase 2: after the intervention).

The Consolidated criteria for Reporting Qualitative research (COREQ) checklist was used when reporting this qualitative study [40]. To ensure trustworthiness, the study followed established criteria for methodological quality, including credibility, transferability, dependability, and confirmability [41,42]. Detailed strategies are provided in S1 Table.

This study was part of a larger study protocol that investigated the effects of a highly intensive functional balance therapy camp in children with DCD, with outcome measures on all levels of the ICF. Further details on the intervention, recruitment, participants, and quantitative outcome measures can be found in the published protocol [34].

### Ethics

This study was approved by the Committee for Medical Ethics of Hasselt University, Belgium (B1152022000001). All parents of the children who participated in the therapy camps received an information letter to inform them about the purpose and design of the study. They gave written consent for their child to participate in the camp and for themselves to participate in this qualitative part of the study.

### Research team

At the time of the study, all members of the research team were researchers affiliated to a Belgian University. MC, IvdV, and BP had extensive experience in conducting, teaching and/or guiding qualitative research. ER, EV and KK brought valuable expertise from their previous roles as promotors on qualitative research projects and through their close involvement in coaching qualitative research. SV was trained by the other team members and followed a PhD training on qualitative research. PM did not have experience in conducting qualitative research. All members of the research team were female, with the exception of ER and PM. Except from meeting several times during the intervention, none of the researchers established a relationship with participants before study commencement. Next to the study’s goal and the researchers’ education and experience, parents were not aware of any other characteristics of the researchers.

### Intervention

A six-day, highly intensive functional therapy camp (40 hours) for children with DCD with a primary focus on balance-related activities was conducted. The camp consisted of six activity categories: jumping, sitting balance, walking and running, circus skills, individual goals, and group activities focused on social interaction. Each category included relevant balance exercises prioritizing enjoyment and collaboration over competition. In the “Individual goals” category, children worked with their individual therapist to practice up to three self-chosen balance-related (e.g., running, skateboarding, cycling, etc.) goals. Three camps were conducted, two in 2023 (April and August) and one in August 2024. Therapy was delivered by physical therapists, maintaining a 1:1 participant-to-therapist ratio, allowing individual adjustments for each activity. All children performed the same tasks, except during “Individual goals” sessions. Both implicit and explicit motor learning strategies were used, tailored to the specific needs of the child based on factors like age, individual abilities, and learning phase, as well as the type of task and environment [43–45]. Although the intervention targeted children, without a separate parent component, parents remained involved through briefings and frequent informal interactions with both the individual therapists and other parents. More information on the intervention (activity categories and key principles) and participant recruitment and selection is presented in S1 File and S2 File respectively. More elaborated information on the intervention and outcome measures can be consulted in the published protocol [34].

### Data collection and sampling

#### Sampling phase 1.

Purposive sampling was used by inviting the parents to participate in this qualitative study face-to-face or via e-mail after their child’s inclusion in the intervention of the first two camps (April and August 2023) (Fig 1). No specific exclusion criteria were applied.

### Data collection phase 1

Two open-ended questionnaires (S3 File) were developed by the research team (SV, IvdV, MC, ER, PM, EV, KK): one to be completed by parents before the start of the intervention on the current situation and, a second one to be completed after the intervention on the perceived changes on participation in physical activities. Questionnaires were distributed immediately after the intervention and could be completed up to sixteen weeks after the intervention. The open-ended questions in both questionnaires were based on experiences shared by the parents after a pilot study with a similar intervention in 12 children with DCD (April 2022, non-published).

The following information was collected from the participants: age, gender, and education level of both parents, number of siblings in the family, and presence of neurodevelopmental disorders in siblings. The open-ended questions in both questionnaires focused on four topics: the perceived changes within: 1. the child, 2. the parent, 3. the family context (defined as interaction between child, parents, and siblings, if applicable) and 4. the social interactions of the child (defined as interaction with peers).

Open-ended questionnaires were completed digitally in Google Forms and extracted in an Excel spreadsheet. They were only considered for analysis when both pre- and post-interventional open-ended questionnaires were completed. The completed open-ended questionnaires were securely stored on the institutional Google Drive with restricted access to ensure data confidentiality. The time between the intervention and the completion of the post-interventional questionnaire was registered. Findings of one questionnaire were unclear due to the mother’s different native language, by exception a follow-up interview was conducted to clarify the open-ended answers.

### Sampling Phase 2

Participants in this study were parents of the children who had participated in the third camp in August 2024 (Fig 1). Sampling, in- and exclusion criteria and the collected descriptive information were the same as in Phase 1.

### Data collection Phase 2

The interview guide (S4 File) was based on the findings of Phase 1, as shown in the mapping table in S2 Table, and developed by the same research team (SV, IvdV, MC, ER, EV, KK). The interview guide underwent two rounds of extensive feedback from the research team and finally included one opening, one transitioning, three key, and one ending question(s), with several suggestions for follow-up questions to elaborate on answers in more detail [46]. The opening question requested a short introduction by each parent and their experience during and after the intervention. The transitioning question requested the perceived changes on daily functioning of the child, parent, family and social interaction (similar to Phase 1). This was done by using word clouds [47] performed in Wooclap and presented live during the focus group. Key questions were about the how the perceived changes were interrelated, within the child and between the child, parent, family and social interaction. Findings of Phase 1 were added by the moderator to facilitate the conversation or check specific answers.

Two focus groups were organized in November 2024. Since data richness and group dynamics are similar in face-to-face or online audiovisual focus groups [48–50] and participants lived in different regions of Belgium, the focus groups were held online to facilitate participation [50]. Participants received the Wooclap link for word clouds and the Google Meet link for the focus group via email one month in advance, with a reminder sent one day before the focus group. To facilitate the group discussion and word clouds, participants were asked in advance to think about the perceived changes in daily functioning of the child, parent, family, and social interaction after the intervention.

The number of participants was limited to a maximum of eight [51]. All participants were informed at the start of the focus group to use the chat and to raise digital hands if they wanted to add something to the discussion. A moderator (MC) directed and facilitated the discussion and invited participants to share their experiences and thoughts [51]. An observer (SV) was present to take comprehensive fieldnotes, check if all key questions were discussed, and summarize the key points of the discussion at the end of the discussion [51]. A third person (KK) was present to keep the time and provide technical support. Aside from the participants, moderator, observer, and technical support, no additional individuals were present during data collection, and no extra interviews were conducted.

Following the focus group, a member check was conducted by sending participants a summary of the discussions, allowing them to provide feedback or add insights. However, none of the participants responded to this. Full transcripts were not shared with the parents. The focus groups were audio and video recorded, and lasted about 90 minutes. Recordings and notes of the focus group were securely stored on the institutional Google Drive with restricted access to ensure data confidentiality. As participants were limited to parents of the participating children, data saturation was not the primary criterion for ending data collection.

### Data analysis Phases 1 and 2

Median age (range), gender, education level of both parents, number of siblings in the family and presence of neurodevelopmental disorders in siblings were reported to describe both cohorts.

A thematic analysis with inductive open coding, as described by Braun and Clarke [52], was used to analyze both study Phases, comprising the following five steps: (1) familiarizing with the dataset through a process of immersion, (2) coding by systematically adding code labels, (3) generating initial themes by identifying shared patterned meaning across the dataset, (4) developing and reviewing themes after checking themes with the total dataset and (5) refining, defining and naming themes [52]. In Phase 2, retroductive reasoning was applied using the fPRC model for Steps 3–5. The analysis was performed by members of the research team and assisted by master students in Step 1–3 (Phase 1: LC, SB; Phase 2: IL, IB).

In Phase 1, both open-ended questionnaires were taken together in the analysis. Step 1 was performed by all reviewers involved (Sv, IvdV, LC, SB). Within Step 2, the first three open-ended questionnaires were analyzed by all reviewers to focus the analyses and to calibrate the coding procedure. The remaining open-ended questionnaires were analyzed with dual coding by two reviewers: one researcher (SV or IvdV) and one master student (LC or SB). After every six open-ended questionnaires, the open codes were discussed in a consensus-meeting between the two reviewers. If consensus could not be reached, senior adjudication was applied by consulting the other first author. Based on these consensus meetings, the coding strategy could be adjusted for subsequent open-ended questionnaires. After analyzing all open-ended questionnaires, all four reviewers (SV, IvdV, LC, SB) shared first thoughts about subthemes and themes (Step 3). In Steps 4 and 5, a meeting with other members of the research team (SV, IvdV, ER, KK, EV) was organized to identify the final themes.

In Phase 2, each focus group was transcribed verbatim by IB and IL and checked by SV. The transcripts were pseudonymized, by removing the identifying information (e.g., names). A thematic analysis with inductive open coding was performed on the verbatim transcripts and consisted of the same steps as Phase 1 [52]. Coding (Step 2) was conducted using a dual coding approach by two independent reviewers (SV and IB or IL) with senior adjudication by consulting IvdV if needed. After coding half of a transcript, open codes were discussed in a consensus meeting in order to reach inter-coder consensus and adjust the coding strategy if needed. Generating initial themes (Step 3) was performed by SV, IB, IL. Developing and reviewing themes (Step 4) and refining, defining and naming themes (Step 5) was conducted in a general meeting with the research group (SV, IvdV, MC, ER, KK, EV). After inductive open coding, subthemes and themes were defined. Based on retroductive reasoning, the underlying relations aligned with the fPRC model as described by Imms and colleagues [11]. Therefore, the model was used to describe relations between identified changes in Steps 3–5, if possible. If necessary, additional subthemes were created and added to the model. The relations between the identified changes were visualized using adaptations to the original fPRC model. The model was obtained and adapted with permission of Prof. Dr. Christine Imms, first author of the original publication [11]. Processes identified in the original model and their definitions can be consulted in S3 Table.

Since the dataset was relatively small and the coding process remained manageable, data analysis was conducted using Microsoft Word and Excel and no specialized qualitative analysis software was employed. Traceability was maintained using dated versions of documents with structured coded sheets for all reviewers and a consensus document with coding decisions. All steps in data analysis were standardized and fully documented by memos with reviewers’ thoughts and completion dates. In this paper, names are pseudonymized for privacy reasons.

All master students involved were educated (eight hours) by the first authors to get familiar with thematic analysis, acquire analyzing skills and verbatim transcribing (only Phase 2). Education included reading literature, watching relevant YouTube tutorials regarding the coding process and practicing coding and procedural techniques in similar open-ended questionnaires (Phase 1) or transcripts of focus groups (Phase 2) of another study on which they received extended feedback.

## Results – Phase 1

### Participant’s characteristics

Parents of all children (n = 23) agreed to fill in the open-ended questionnaires. Parents of one child only filled in the pre-interventional questionnaire and were therefore excluded from the analysis. This resulted in a total of 44 open-ended questionnaires included for analysis. All pre- and post-interventional open-ended questionnaires were filled in by the mother, with exception of two pre-interventional and one post-interventional open-ended questionnaires that were completed by both parents. Descriptive data of the sample are shown in Table 1. Descriptive characteristics of the child sample of Phase 1 is presented in S2 File.

**Table 1:** Descriptive details of sample Phase 1.

	N		n	
**Number of included families**	22			
**Descriptive details provided (yes:no)**		16:6		
**Median (range) age of parents (years)**	32	41 (37 – 52)	18	39 (37 –52)
**Sex of parents (F:M)**	32	16:16	18	16:2
**Parental education level**	32		18	
Secondary education		2		1
Associate degree		3		0
Bachelor degree		15		9
Master’s degree		11		7
Doctorate degree		1		1
**Number of children in the family**	16			
1 child		2		
2 children		9		
3 children		3		
4 children		2		
**Comorbidities in siblings (yes:no)**	14	10:4		
Combined DCD + autism		1		
DCD		2		
Hearing loss		1		
**Timing of post-intervention completion**	22			
<4 weeks after intervention		10		
4 −8 weeks after intervention		6		
8-12 weeks after intervention		3		
>12 weeks after intervention		3		

N: total sample of parents, n: subsample of parents who completed the open-ended questionnaires; F: Female; M: Male; DCD: Developmental Coordination Disorder; autism: Autism Spectrum Disorder

### Changes in participation in physical activities after intervention

**Fig 2:**
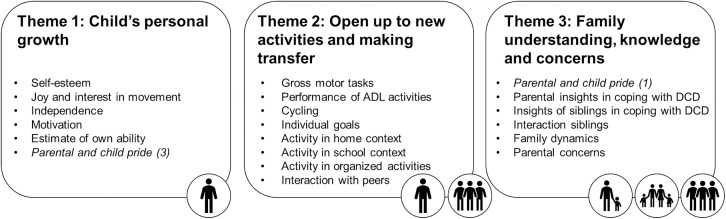
Overview of defined themes and related subthemes of Phase 1. NOTE. Pictograms indicate which members of the child’s environment are involved: child, parent, family, or social interactions. Cursive subthemes appear in multiple themes, as indicated by the number. ADL: Activities of Daily Living.

Changes in participation in physical activities were reported on multiple facets. After data analysis, three major themes on detected changes were identified: (1) Child’s personal growth; (2) Open up to new activities and making transfer, and (3) Family understanding, knowledge and concerns. Fig 2 shows an overview of subthemes and themes.

### Theme 1: Child’s personal growth

This theme concerned changes in personal growth of the child and consisted of six subthemes: (1) Self-esteem; (2) joy and interest in movement; (3) Independence; (4) Motivation; (5) Estimate of own ability and (6) Parental and child pride (Fig 2).

Almost all parents indicated that their child gained more self-esteem after intervention. For example, the mother of Kyle (6 years old) said *“He has gained more self-esteem since the camp. He now says: At the circus camp, I have learned that I can do it”.*

Additionally, parents observed that the children had developed more joy and interest in movements:

“*He [Jonathan, 10 years old] slides and dances through the house. Also, he moves more confidently in soccer and judo.”*

Some parents also mentioned more motivation for physical activities with for instance the mother of Lucas, 8 years old, saying “*He looks forward to his sports day now, which was a horrible day before.”*

A lot of parents shared how proud their child was of themselves. The mother of Sarah, 7 years old, stated: “*She was so proud during the performance, we had to show the recordings to the rest of the family. The joy she radiated there was immense.”*

In addition, few parents also indicated that the child had become more independent after the camp, both in motor skills and in daily life activities: “*We need to offer less help, as he [Tom, 11 years old] is now more likely to try things on his own or ask for help less frequently.”*

Lastly, few parents did mention that their child was able to make a better estimation of their own abilities with Sophie, mother of Dave (6 years old), saying *“I think Dave has already received a noticeably better estimation of his motor abilities since the camp. Based on the experiences he gained there, I believe I can see an improvement in how he estimates himself.”*

### Theme 2: Open up to new activities and making transfer

This theme concerned changes in the performance of activities and the interest in trying new activities by the child and consisted of eight subthemes: (1) Gross motor tasks; (2) Performance of activities of daily living (ADL); (3) Cycling; (4) Individual goals; (5) Activity in home context; (6) Activity in school context; (7) Activity in organized activities and, (8) Interaction with peers (Fig 2).

Improvements were mainly observed in gross motor skills, while fine motor challenges persisted for most children. The mother of Karen (8 years old) mentioned: “*Before camp, she couldn’t jump rope, but she’s made significant improvement since.”*

A few parents indicated improvement in ADL activities that were not specifically targeted during the camp, such as tying shoelaces. One parent stated: *“My son [David, 9 years old] appears to have become quicker in many daily tasks, such as using the toilet, getting dressed, and preparing for school.”*

Cycling was a commonly chosen individual goal. Multiple parents reported that their child dared to join more cycling outings, exhibited greater enthusiasm for cycling, could cycle longer distances, and that they had fewer concerns regarding traffic situations involving cycling. The mother of Tom (11 years old) noted: “*Cycling is much better. Much more stable, less fearful, much more confident.”*

Similar improvements were reported in other individual goals. The mother of Peter (12 years old) noted: “*Skating is much more controlled, sturdy, stable, with him really applying the techniques he learned from his instructor.”*

In the home context, some parents reported that their child engaged more in games with siblings, participated in more adventurous and sports activities as a family, and spent more time playing outdoors. “*During our long weekend in Austria, we were able to do more active and adventurous hikes than before! However, we still take into account the fact that he [Lucas, 8 years old] may stumble.”*

Several children dared to engage more in sports activities on their own. “*She [Karen, 8 years old] spends more time on the playground and is less likely to turn down invitations to play tag, for example.”*

Four children showed interest in starting a new hobby after the intervention, with two having already started the new hobby at the time the parent completed the questionnaire. The mother of Julie (8 years old) stated: *“Since the camp and especially after learning the cartwheel and handstand, she is now very eager to start gymnastics. We’re going to try it out.”* Other parents also noted that their child experienced more enjoyment in existing hobbies.

Many of the children already had good social interactions with their peers before the intervention, so little to no change was observed by the parents in this regard. However, a few parents did mention that their child now dared to participate more in group games on the playground. “*He [Kyle, 7 years old] participates more in running games on the playground.”*

### Theme 3: Family understanding, knowledge and concerns

This theme concerned the environment of the child including parent, family context and social interaction. It consisted of six subthemes: (1) Parental and child pride; (2) Parental insights in coping with DCD; (3) Insights of siblings in coping with DCD; (4) Interaction siblings; (5) Family dynamics, and (6) Parental concerns (Fig 2).

Several parents mentioned gaining a better understanding of DCD and the need to show more understanding for their child’s motor problems. Through the camp, they saw that their child was not the only one who struggled with motor skills. Marie, the mother of Lynn (6 years old) said:

“*Also, I have noticed that she can accomplish a lot, which reassures me. She mainly needs* *good guidance and especially more practice time… I need to patiently guide her in learning something new, possibly breaking it down into smaller steps. Sometimes, I got angry because she seemed unwilling to understand. I think I need to show more understanding and ask her more questions instead of directing her on how to do things”*

Overall, parents got insights in how to cope with their children’s motor difficulties. This was also observed in siblings. *“His [Ian, 9 years old] sister suddenly understood that her brother sometimes has to try harder to accomplish something. I feel like she’s also less angry when things go wrong now”*

This resulted in better family dynamics with Margot, the mother of the 7-year-old Casper, saying: “*I’m encouraging the kids to try things more often and to be less protective. The youngest also has some motor challenges, so I might push them a bit more to try instead of occasionally holding them back.”*

Few parents also mentioned more interaction between siblings “*He [Jef, 7 years old] seems more excited to interact with his older brother, appearing noticeably more confident.”*

About half of the parents experienced fewer concerns, including less concerns about motor skills, social status, trying new things with the family, and cycling. The mother of Lucas (8 years old) stated: *“Looking ahead, I see Lucas continuing to make great progress. He may not become an athlete but he enjoys the activities he takes part in and that’s what truly matters. Our focus will be on maintaining the self-confidence and enjoyment he gained during this camp well into the future.”*

However, many parents reported ongoing concerns especially related to clumsiness in daily life activities, independence, cycling, interactions with siblings and peers, and worries about high school.

*“In terms of gross motor skills, I am now reassured that he [Casper, 7 years old] can keep up well. The fact that he can now reach out his hand while cycling gives me confidence that his balance is improving. However, I notice that there is still a lot of work to be done in terms of ball skills and similar aspects. On the whole, things haven’t changed much in that regard. My concerns about clumsiness in daily activities (dressing, eating, etc.) and gymnastic classes from before the camp still persist.”*

## Results – Phase 2

### Participant’s characteristics

Parents of all children (n = 12) agreed to attend the focus groups, however parents of one child were not able to join the focus group due to practical reasons. Fourteen parents (three fathers) attended the focus groups, equally divided between both groups. If both parents of a child were present, they had similar experiences. Descriptive data of the sample can be consulted in Table 2. Descriptive characteristics of the child sample of Phase 2 is presented in S2 File.

**Table 2:** Descriptive details of sample Phase 2.

	N		n	
**Number of included families**	12			
**Descriptive details provided (yes:no)**		11:1		
**Median (range) age of parents (years)**	21	41 (32 - 55)	14	40 (32 –47)
**Sex of parents (F:M)**	21	11:10	14	11:3
**Parental education level**	21		14	
Secondary education		5		1
Associate degree		2		2
Bachelor degree		8		6
Master’s degree		4		3
Doctorate degree		2		2
**Number of children in the family**	11			
1 child		2		
2 children		6		
3 children		3		
4 children		0		
**Comorbidities in siblings (yes:no)**	9	1:8		
Combined DCD + autism		1		

N: total sample of parents, n: subsample of parents who joined the focus groups; F: Female; M: Male; DCD: Developmental Coordination Disorder; autism: Autism Spectrum Disorder

### In-depth exploration of the perceived changes as identified in Phase 1 and how these changes are interrelated

Participants shared the parental perspectives found in Phase 1 and even mentioned some additional changes. First, Parents not only experienced **more acceptance of the DCD diagnosis** themselves, but also in their children. “*For him [Vince, 9 years old], the camp was a significant step in the process of accepting DCD, as it made him feel good to be among other children and to realize,”I’m not the only one.”* Second, the **research context** of the intervention was mentioned by some parents as beneficial. The father of Robin, 7 years old, mentioned: *“The research context offers additional support to the environment, complementing existing resources such as physiotherapy and school-based support, and thereby creating greater understanding.”* Third, participants in Phase 2 also described **increased understanding and knowledge** in their children with DCD and broader family and friends. The mother of Nora, 9 years old, stated: *“The grandparents now recognize the seriousness of DCD, having seen firsthand that these children share similar characteristics and that the challenges they face are real.”*

**Fig 3:**
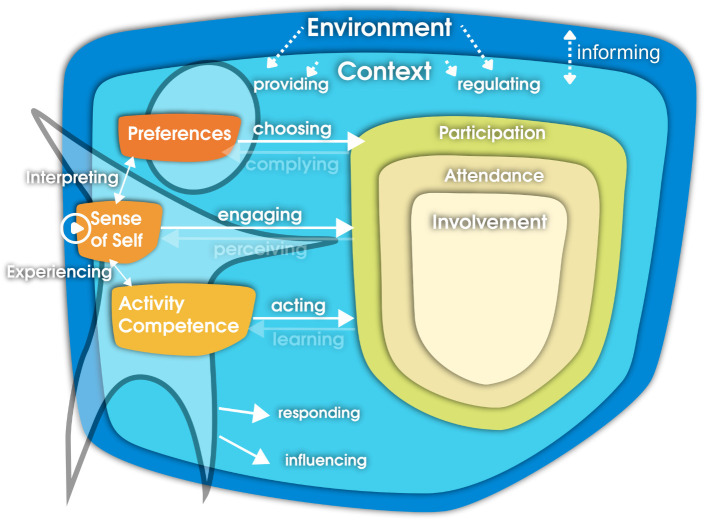
Findings of Phase 2 indicated on the fPRC model. NOTE. Adapted fPRC model showing relationships between post-intervention changes. Thick arrows represent reported effects. Solid arrows indicate (targeted) child-related relations; dotted arrows indicate outcomes related to non-targeted individuals. The play icon indicates the onset of the processes referring to “Onset” which was added to the original model along with “Informing”. Adaptations to the model were visualized with permission from Prof. Dr. Imms, first author of the original publication [11].

After data analysis, there were no subthemes related to the processes of “Complying,” “Perceiving,” and “Learning” of the fPRC model. In addition, two extra processes were added to the model: “Informing,” meaning any communication initiated by parents directed toward the child’s environment, and “Onset,” describing how observed changes began and how they were interrelated according to parents. Quotes regarding these processes can be found in Themes 3 and 4 respectively. Fig 3 shows the findings of this study on the fPRC model. Two types of processes were identified: expected processes, which were directly related to the child as the primary target of the intervention, and unexpected processes, related to individuals other than the child and were not explicitly targeted by the intervention.

**Fig 4:**
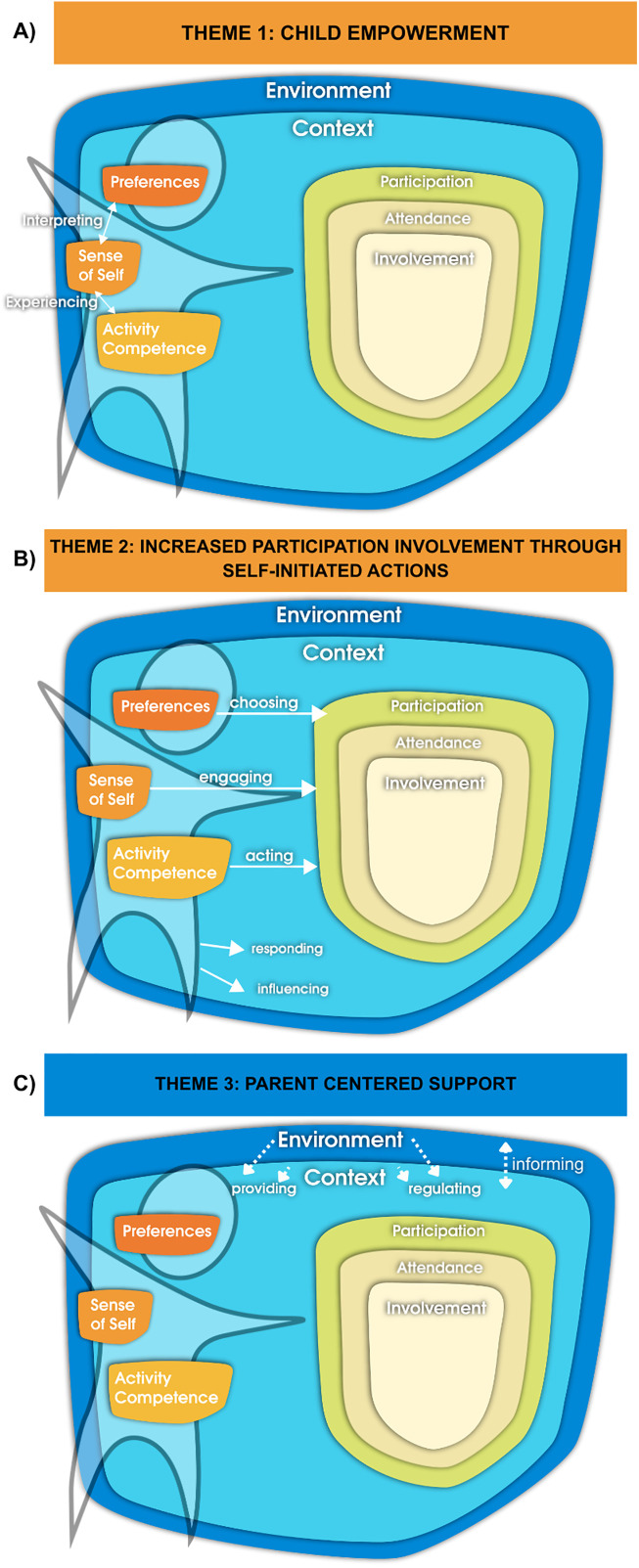
Themes in underlying relations between changes in child participation. NOTE. Themes on the adapted fPRC model showing relationships between post-intervention changes in child participation. Solid arrows indicate (targeted) child-related relations a; dotted arrows indicate outcomes related to non-targeted individuals. The process of “Informing” was added to the original model. Adaptations to the model were visualized with permission from Prof. Dr. Imms, first author of the original publication [11].

Regarding the relations and their onset, four themes were identified: (1) Child empowerment; (2) Increased child involvement through self-initiated actions; (3) Parent centered support and, (4) Empowerment-induced sustained changes after three months. An overview of subthemes and themes is shown in Fig 4.

### Theme 1: Child empowerment

The subthemes within this theme describe the processes within intrinsic factors of the child and include the processes of: (1) Interpreting; (2) Experiencing and (3) Self-regulation (Fig 4a).

Regarding “Interpreting,” parents mentioned that the intervention improved the child’s sense of self and thereby influenced the child’s development of preferences.

*“Before the camp, the child was hesitant to try difficult things. -At camp, he [Leo, 7 years old] experienced a shift: ‘Once I learn something, I get better at it and it becomes more fun.”*

The subtheme ”Experiencing” clearly showed improved sense of self due to success experiences during the intervention which persisted in other contexts (home, school etc.) after the intervention. *“Her [Nora, 8 years old] self-confidence came from learning things she once thought she couldn’t do, or that she wouldn’t succeed at, or wouldn’t succeed in quickly.”*

”Self-regulation” was a subtheme that was highlighted frequently by parents. They mentioned an adapted level of reasoning after the intervention where children show more acceptance with their diagnosis and situation demonstrating the presence of inner processes. The mother of Kevin, 7 years old, mentioned: *“Last year, my son would shrink into his shell at the playground or feel sad watching the fast and strong children. He now recognizes that his strength isn’t physical, it’s verbal.”*

### Theme 2: Increased participation involvement through self-initiated actions

The subthemes describe an active role of the child with both the context and environment: (1) Influencing and (2) Responding, as well as in participation contexts with (3) Choosing, (4) Engaging and (5) Acting (Fig 4b).

Parents stated that their children impacted and responded more actively to their environment. Children set their boundaries more actively and request for help, both spontaneously and in situations where they experience difficulties. The mother of Theo, 8 years old, stated: *“When things get a bit too difficult for him, he now dares to say, ‘Yes, but Mum, I have DCD, I can’t just do that.’ He’s confident enough to say it as if it is the most normal thing in the world.”*

Also in participation situations, the child acted more actively. Children performed more activities because they felt more competent when doing them. Additionally, they enjoyed them more and therefore chose to perform certain activities more frequently or to learn new activities. The father of Robin, 7 years old, said: *“My son could already ride a bike, but he practiced a lot during the intervention. Now, he rides to school almost every day and goes to music lessons. He rides with us, and you can see how much more confident and skilled he has become.”*

The increased personal growth in Phase 1 resulted in more participation involvement. Parents mentioned that their children are more involved in new and already existing participation situations after the intervention. The mother of Louis, 10 years old, mentioned *“During the camp he learned how to inline skate, something he truly wasn’t able to do before. Mastering it gave his self-confidence such a boost that he’s now very enthusiastic and even wants to try ice skating this winter.”*

### Theme 3: Parent centered support

Parents also experienced changes in their own behavior and interactions with their child with DCD, both conform the fPRC [11], resulting in: (1) Providing, (2) Regulating and (3) Informing, which was added to the fPRC (Fig 4c).

In line with the findings from Phase 1, parents experienced having more insights in DCD but also acted differently towards their child in participation situations. Parents felt more confident and introduced more practice opportunities after the intervention. *“After the camp, I finally felt confident enough to give my son [Theo, 8 years old] a knife at dinner. Before that, I was always afraid he might cut his fingers and would take the knife away as a precaution….The intervention also gave me self-confidence, and then I was like, ‘Let’s try this.’ Now, he helps in the kitchen and dares to cut things for us.”*

Furthermore, they mentioned that the intervention experience helped them to manage the challenges in difficult situations by accepting their children’s motor difficulties and coping better with them. As a consequence, the interaction with their child changed after the intervention. The father of Louis, 10 years old, said: *“What we’ve noticed is that we encourage him more often, because he can do more than we thought …which is why we often say, ‘Try it anyway.’”*

Parents described information exchange within different levels of the environment (e.g., school, hobbies). After the intervention, parents had greater awareness of their children’s motor difficulties resulting in feeling more confident and faster requesting more help to other members of their child’s environment. The mother of Vince, 9 years old, said: *“I now have more self-confidence to talk about DCD, because before, it felt a bit theoretical. For example, I’ve mentioned the camp a few times, at the new school and at the music academy. I feel it helps raise awareness, as in, ‘Oh, there’s more to this than we thought, it’s really a thing,’ and they start looking into it. At the new school, the entire team will even receive training on DCD.”*

### Theme 4: Empowerment-induced sustained changes after three months

Since changes are experienced over all categories of the fPRC, this theme describes the onset of changes with one subtheme: (1) Onset.

Parents indicated that specific features of the intervention created an accessible environment for learning motor activities. They mentioned that the fun atmosphere and individual therapists facilitated immediate success experience during activities, or created dialog between therapist and child in case of failure. The fact that children joined a full week of intervention reduced time pressure and allowed them to progress at their own pace. According to some parents, this was sometimes not the case in private therapy settings. The mother of Tara, 8 years old, said: *“My daughter benefited from the buddy system, which gave her the confidence to try many new things. The camp’s warm, playful environment, filled with games, ice cream, and fun, created a relaxed, non-therapeutic atmosphere. This made her feel safe and motivated in a way she hadn’t experienced before, even with her physiotherapist or speech therapist.”*

However, for some parents it was difficult to define a specific starting point, but they did mention that changes tend to trigger other changes. *“I believe the changes experienced are indeed multi-directional, involving us, our son [Vince, 9 years old], and the school/hobbies, with each aspect reinforcing the others.”*

Overall when combining these insights of the “Onset” with findings of themes 1–3, it can be assumed that specific characteristics of the intervention (e.g., highly intensive camp model, individual guidance, fun atmosphere) initiated a domino effect. This resulted in increased child empowerment, in both motor and non-motor tasks, and changed their behavior in participation contexts and interactions with people in their environment.

The mother of Nora, 8 years old, said: *“Both in the playground and in the pool, my daughter now automatically tries things she wouldn’t have attempted before. She has also started new hobbies, something we used to really have to push her to do.”*

At the same time, increased parental confidence influenced how they communicated and behaved with their child in daily life and with other people in their child’s environments (e.g., school, hobbies, broader family context) starting a positive cycle that created a supportive learning environment for the child and potentially helped to induce sustained changes in a child participation in physical activities.

## Discussion

This exploratory sequential, multimethod qualitative study aimed to explore parents perspectives on their child’s participation in physical activities after a highly intensive functional balance training. In the first phase, open-ended questionnaires were used to assess perceived changes in child participation in physical activities. After this Phase, focus groups were performed in a second phase to gain more detailed insights into the changes of Phase 1 and how these changes are interrelated.

The study resulted in three main findings. First, the focus groups confirmed the results of Phase 1, with parents reporting changes in their child’s personal growth, opening up to new activities and making transfer to other tasks and greater understanding of DCD among parents and siblings. Additionally, the focus groups revealed new insights, including the impact of the research context, increased acceptance of their child concerning their DCD diagnosis and increased insights of DCD of their child and extended family and friends. Furthermore, the focus groups revealed in-depth insights on how these changes interrelate, highlighting child empowerment, increased participation involvement through self-initiated actions and increased parent-centered support. Secondly, while changes at child level were expected, as the intervention directly targeted children, the high number of positive changes in parents, families, and social interactions were remarkable since these were not directly addressed by the intervention. Lastly, this qualitative study offers in-depth insight into how these changes were initiated. Parents described the changes as beginning with a domino effect, where specific aspects of the intervention design (e.g., individual guidance, the highly intensive group format, and a fun atmosphere) served as key facilitators of their child’s motor learning process and empowerment in both motor and non-motor domains. Subsequently, a cyclical process emerged as parents gained confidence, their communication and interactions with their child, as well as with schools and other environments, changed. This empowerment created more learning opportunities which in turn enhanced the child’s self-esteem and motivation to practice. In this way, this potentially contributed to sustained changes in their child’s participation after three months.

These findings partly align with findings of earlier highly intensive CO-OP interventions for children with DCD where both parents and children reported increased understanding of the disorder [23], improvements in the child’s attitude, motivation [24] and greater confidence to try motor activities [23,24]. However, unlike a previous study which found no significant changes in children’s overall participation [23], parents in the current study reported clear improvements across multiple participation domains, extending beyond the physical domain. This discrepancy may be attributed to differences in intervention design. While both programs were highly intensive and tailored, the other interventions only partly (6,5 [23] to 9,5 hours [24]) involved individual sessions where the current intervention provided each child with an individual therapist throughout the complete 40-hour program focusing on individualized learning processes and experiencing motor successes. Parents identified this personal approach and repeated success experiences as a key factor in their child’s empowerment. The positive impact of individually tailored interventions is also supported by the systematic review of Adair and colleagues reporting improvements in child participation after tailored interventions [36]. However, they reported larger intervention effects when the primary intervention goal is related to improving participation and smaller effects when it is assessed as a secondary outcome [36]. This contrasts with our findings, which showed impact on participation, even though it was not the primary aim of the intervention. In the applied intervention, the individualized approach focused on the experience of motor success by using mainly implicit learning principles with child-specific tailoring based on child factors (age, learning phase, personality), type of task and environment [34]. Previous research indicates that errorless motor learning interventions targeting fundamental motor skills can enhance enjoyment of participation in physical activities in children with DCD [53]. Providing an intervention that is focused on experiencing motor successes can accommodate the motor challenges these children are facing and promote feelings of success and enjoyment in motor activities [53,54].

Parents reported increased participation involvement in their children after the intervention, both in activities directly related to therapy (e.g., cycling) and in activities which were less related to the intervention context (e.g., dressing). According to the fPRC, attendance is also a key component of participation. However, since attendance is a prerequisite for involvement, improvements in participation involvement suggests that attendance was sufficient for involvement to occur. This is a promising outcome given that children with DCD typically show significantly reduced participation involvement, particularly in school and community settings, compared to their typically developing peers [3,35]. Furthermore, parents of children with DCD reported more environmental barriers, less environmental helpfulness and less overall environmental support compared to typically developing children [3]. Moreover, environments of children with DCD are often not adapted to their specific needs, such as classrooms or sports groups managed by a single adult responsible for all children [3] resulting in less time on task, less experience of motor successes, etc. The findings of this study suggest that when adapting the environment to promote optimal learning, e.g., by experiencing motor successes, individual tailoring and increased therapy time, children can improve their participation involvement in physical activities. This highlights the importance of shifting the focus from solely improving motor skills to also recognizing and incorporating strengths, creating and celebrating successes and thereby increasing a sense of belonging and participation involvement.

Current guidelines for children with DCD recommend goal-oriented therapy situated on the activity and participation levels of the ICF by incorporating task- and context-specific elements [1]. The intervention in this study aligned with these recommendations, and the qualitative findings highlight their relevance. While guidelines highlight the role of support from parents, teachers, and significant others in the child’s environment to experience treatment success [1], our results emphasize that such environmental support is not only essential for short-term gains but also crucial for sustained changes in the child’s participation in physical activities, exceeding treatment goals. Although the intervention did not specifically target parents (e.g., coaching of parents was not part of the study), they reported changes in their support toward the child and in their communication with the environment. It can be assumed that these changes would increase even more if parents were specifically targeted in the intervention. For instance a mixed-methods study by Camden and colleagues examined the impact of an online educational module on parental knowledge and behavior in relation to their children with DCD [55]. The study demonstrated clear improvements in parents’ understanding translating into changes in their behavior with their child and in their communication with others in the child’s environment (e.g., school, physicians) leading to beneficial adjustments in daily routines for the child [55]. Remarkably, the motor intervention used in this study also led to similar changes in parental behavior toward both the child and their environment, even without including educational modules for parents. This may be attributed to the intervention’s highly intensive nature, which required frequent parental presence, interactions with therapists and other parents, and direct observation of their child’s progresses. These findings suggest that parents can acquire and apply relevant knowledge through intensive involvement, integrating it into daily life. However, equal to tailoring therapy to children, there is not one correct way of involving parents since it is dependent on the parent. It is possible that some parents need more structured, explicit educational support, as provided in the study of Camden and colleagues [55].

Next to involving parents in their child’s therapy, the cyclical process suggested the fact that parental empowerment is key in stimulating their child’s learning processes. This suggests that therapy should not only focus on the child but also actively focus on the parents. The education of caregivers, in order to achieve changes in child participation, has been gaining attention over the past years with multiple evidence-based therapeutic approaches focusing on implementing education for caregivers in pediatric rehabilitation [56–60]. One example is the partnering for change model in school-context, in which therapists work together with teachers and parents in order to facilitate learning in specific school context but also to empower the teacher and parents when therapists are not present and change the life and daily environment of the child [56]. Another example is provided in Pathways and Resources for Engagement and Participation (PREP), a therapy model where therapist actively involve parents and family to learn individual goals [57,58] and proven to enhance overall participation in children with physical disabilities [57]. These programs demonstrate that it is possible to prioritize and engage caregivers, serving as models for future initiatives for children with DCD.

The highlighted importance of parents in enhancing child participation, both in this study as in previous literature, suggests that parents should have a more prominent role in the fPRC model [11]. One subtheme captured the interactions among multiple caregivers, beginning with the parents. While the original fPRC model includes a specific participation context and broader environmental factors—such as family, leisure activities, and school environment [11] — our findings show that additional inter-environmental processes are present and can actively enhance participation but mainly start from the parents. For instance, parents reported feeling more confident and initiated communication with the school about their child’s DCD and associated challenges. In response, the school organized a lecture on DCD for all teachers, raising awareness on DCD. This suggests that it could be relevant to expand the fPRC model by including additional layers of environments, as seen in other models such as the Ecological Systems Theory of Bronfenbrenner [61]. This theory describes different, related environmental systems impacting a child’s development [61]. Our findings similarly showed that these environmental systems influence child participation, supporting the inclusion of extra environmental layers in the fPRC model.

### Strengths and limitations

To our knowledge, this is the first qualitative study that explored interventional effects on child participation in physical activities from the perspective of parents of children with DCD. By first analyzing data from the open-ended questionnaires, the focus groups could be tailored to these insights, with more targeted questions allowing for a deeper understanding of how these changes are interrelated. However, this approach might also be a possible limitation as both phases were conducted in different cohorts. Even though the perceptions of the second cohort were explicitly explored, it remains possible that parental perceptions differed from the first cohort. As differences between cohorts may have subtly shaped how themes were identified and understood, this could limit direct transferability between cohorts and potentially affect the generalizability of findings. Nevertheless, both cohorts were broadly comparable in demographic characteristics (median age 40 years old, majority mothers, and most parents holding bachelor’s or master’s degree) and family composition (two or three children), which supports the transferability of findings between cohorts.

Focus groups involve group dynamics and emotional contexts in which emotional biased, conformity biased, or social desirability answers may be given. However, this is inherent to this type of data collection as the purpose of focus groups is to listen and gather information. It is a way to better understand how people feel or think about an issue, product, or service [51,62]. However, in order to minimize these type of biases, the focus groups were conducted in small groups and the moderator had special attention for the group dynamics by starting the focus group with a general introduction including the rules of conduct. During the focus group the moderator responded to non-verbal signals, actively stimulated less talkative parents and prevented dominant parents from dominating the discussion. Nevertheless, there is a small possibility that parents felt hindered in sharing their experiences fully and authentically.

Furthermore, reflecting on the study design and findings, it would have been beneficial to incorporate the fPRC model from the start, using it as a guiding framework in Phase 1 as well. This may have strengthened the integration and transferability of findings across both phases of the study. Although literature describes definitions of the elements of the fPRC model and provides recommendations on how to use the model, researchers’ interpretations of the model might have influenced the qualitative analysis, potentially resulting in missed or overlapping themes which hinders interpretation.

Also, since the number of participants was limited, generalization of the findings to all children with DCD might be limited. Additionally, the findings should also be interpreted with caution due to potential self-selection of more engaged parents. Parents who voluntarily subscribed their child to the intervention, were likely more motivated and proactive to address their child’s motor challenges. Furthermore, the education level in both cohorts was higher than in the general Belgian population [63]. Therefore, findings may reflect the perspectives of highly engaged and educated parents rather than a broader, more diverse group. Together with potential differences in other demographic characteristics, this might limit the generalizability of findings. Another limitation is that the children’s perspectives were not collected, even though some children were old enough to provide their own views. This is particularly relevant for topics such as social interactions, where parents may have limited insight. Furthermore, by only questioning the parents, potential reporting bias might be present. Finally, a possible immersion effect should also be considered as the unique context of the intervention (1:1 therapist:child and highly supportive environment) differs substantially from daily life. Parents’ perceived improvements may therefore be influenced by this context rather than the content of the intervention alone, which limits the generalizability to everyday settings.

### Implications for further research

As our explorative study showed the potential for involvement of parents to induce sustained changes in their child’s participation after three months, future research should further investigate the impact of caregivers involvement and education to enhance the participation context. Specifically, studies should explore how these factors influence parental perceptions of their child’s motor abilities and the child’s motor behavior and participation. Next to the impact of parental knowledge and empowerment as influencing environmental factors on child participation, a broader focus on more specific environmental factors (e.g., socio-economic background) were not taken into account independently as this was not the focus of this exploratory study. However, it would be interesting to explore this in future research. Qualitative studies with more robust designs, larger samples including both parents and children, reached data saturation and longer follow-up periods are needed to investigate changed perspectives on child participation after motor intervention to fully understand the impact of intervention and the sustainability of the intervention effects. To achieve triangulation of findings, children’s physical activity should be supplemented by additional observational data or teacher reports. In addition, future studies should include a control group to determine whether the observed effects can be attributed to the intervention alone or are influenced by other environmental factors. They should also incorporate objective quantitative measures measuring participation to support or challenge the reported qualitative changes. Lastly, it is suggested to use the fPRC model as a deductive framework from the outset and throughout the entire process to ensure strong interpretive consistency.

## Conclusion

Parents indicated that their child’s participation in physical activities changed after a highly intensive functional balance training. Parents reported changes in their child’s participation, observing greater empowerment of the child and more self-initiated actions within different participation environments, which potentially led to increased participation involvement. However, unexpectedly, parents reported feeling more confident and demonstrated a greater understanding of DCD, which resulted in changes in their behavior and communication, not only towards their child but also in interaction with other environments concerning the child (school, hobbies, etc.). These changes in parental empowerment were key in achieving sustained changes in the child’s daily routines and child’s participation in physical activities after three months. These findings are in line with earlier recommendations and highlight the importance of addressing not only motor outcomes in children with DCD, but also involving and educating caregivers in therapeutic processes to support sustained changes in their child’s participation.

## Supporting information

S1 File Additional information on the intervention. (DOCX)

S2 File Details on recruitment, selection criteria and descriptive characteristics of child sample. (DOCX)

S3 File Open-ended questionnaire pre- (A) and post-interventional (B). (DOCX)

S4 File Interview guide used during focus groups. (DOCX)

S1 Table Overview of trustworthiness components and applied strategies across data collection and analysis ✔ : applied;✗: not applied; ✔/✗: partially applied, -: not applicable. (DOCX)

S2 Table Mapping table how Phase 1 informed Phase 2 DCD: Developmental Coordination Disorder. (DOCX)

S3 Table Definitions of processes among constructs described by the family of participation related constructs [11]*environment-focused processes are briefly described in the publication of Imms et al. [11], definitions are those defined and used by the authors of this study. (DOCX)
