# Supplementary material for: Parental perspectives on the changes in their child’s participation in physical activities after a highly intensive functional balance training for Developmental coordination disorder: A sequential multimethod qualitative study
Source: PLoS One. 2026 May 14;21(5):e0331994. doi: 10.1371/journal.pone.0331994 (PMC13175460; doi:10.1371/journal.pone.0331994)
Supplement: S1 File — (DOCX) [file pone.0331994.s001.docx]

**S2_File: More information on the intervention**

An example of a day schedule is presented in Table 1. Elaboration on the activity categories, dosage and examples for each activity category are presented in Table 2. Operationalization of key principles of the intervention are presented in Table 3.

**Table 1:** example of day schedule

| **Time** | **Activities** |
| --- | --- |
| 8:00 - 9:00 | Pre camp care |
| 9:00 - 9:45 | A1: Balance bikes  (C: Sitting balance) |
| 9:45 - 10:00 | Break |
| 10:00 - 10:45 | A2: Individual goals  (C: individual goals) |
| 10:45 - 11:00 | Break |
| 11:00 - 11:45 | A3: Circus barrel walking (C: Circus) |
| 11:45 - 12:45 | Lunch break (passive) |
| 12:45 - 13:30 | A4: Airtrack jumping games  (C: Jumping) |
| 13:30 - 13:45 | Break |
| 13:45 - 14:30 | A5: Parcours with obstacles  (C: group activities) |
| 14:30 - 14:45 | Break |
| 14:45 - 15:30 | A6: Obstacle relay with dual tasks  (C: walking and running) |
| 15:30 – 15:45 | Break |
| 15:45 – 16:30 | A7: Rope jumping game  (C: Jumping) |
| 16:30 – 17:00 | Relaxation + day closing |
| 17:00 – 18:00 | After camp care |

Legenda: A = Activity; C=Category

**Table 2.** Activity categories, dosage and examples

| **Activity category** | **Description** | **Examples** |
| --- | --- | --- |
| **Jumping**  7/40 hours | Activities involve jumping in a broad context and different sensory situations (stable and unstable surfaces), unipedal and bipedal jumping, with and without different dual tasks (cognitive, motor, auditive). | - Personate different animals while jumping on a trampoline. - Relay race on an airtrack on one leg while keeping a balloon in the air. |
| **Sitting balance**  9/40 hours | Activities are divided in static and dynamic sitting balance. Static sitting balance activities are performed on the ground, stools or benches with a stable or unstable surface and without backrest. While children sit, games, crafting, cooking activities are performed.  In the dynamic sitting balance activities children sit on a moving surface such as sitting on a moving burlap bag or riding a balance bike (unicycle with small steering wheels). Dual tasks (cognitive, motor, auditive) are added to increase complexity. | Static   - Sitting on a gymball and crafting juggling balls. - Cutting vegetables while sitting on a bench without backrest.   Dynamic   - Performing a complex trail on a balance bike while catching balls that are thrown unexpectedly. - Sitting on a burlap bag that is pulled by the individual therapist and remember puzzle pieces. |
| **Walking and running**  6/40 hours | Activities consist of games where children have to reach goals by walking and running. This can be on stable or unstable surface, eyes open or closed, with or without dual tasks, forward, backward or sideward etc. | - Relay with different running activities. Children have to remember puzzle pieces at the end to reconstruct a puzzle. - Perform a complex trail backwards. |
| **Circus**  6/40 hours | Activities include specific circus activities and are performed individually, in duo or in group. Manual circus activities, such as plate spinning or juggling, can be performed as a dual task. | - Barrel walking, if possible combined with throwing a ball. - Stand or walk on a firm circus ball. |
| **Individual goals**  6/40 hours | Children choose up to three individual balance goals before the camp. Therapy starts from task performance analysis and is individually adapted using motor learning principles. | - “I want to learn rope skipping”. - “I want to learn how to inline skate”. - “I want to learn how to ride a bicycle”. - “I want to ride my bicycle more safely in traffic”. |
| **Group activities focused on social interaction**  6/40 hours | The focus is on social interaction, children have to work together to reach goals or win games. | - Perform a complex and unstable trail with the whole group. - Playing tag and remember memory cards at the same time. |

**Table 3.** Key principles of the intervention

| **Principle** | **Explanation** |
| --- | --- |
| Accessible environment | The interventions were organized in a spacious school environment equipped with child-appropriate materials, creating a warm and child-friendly atmosphere. Communication with the organization was easily accessible for both parents and children, all children were familiar with all therapists and organizers and vice versa. Via the buddy system, each child was assigned an individual therapist. This 1:1 connection created strong relationships between the children and their therapists, facilitating an open and comfortable atmosphere in which children felt safe to share their emotions and experiences. These could be both positive and negative. |
| Personal approach | **The individual approach was implemented on several levels. First, during the activities, each child was guided by their own therapist. The activities were designed to allow individual adaptations while still participate in group. Next, during the individual goal sessions, children practiced their personal goals one-on-one with their therapist, creating a strong therapeutic connection. Finally, each child followed their own intervention schedule. Although all children participated in the same activities by the end of the intervention, their weekly schedules could differ depending on whether activities were conducted in small groups, large groups, or individually with their therapist.** |
| Fun atmosphere | **Having fun while moving was the main objective of the intervention.** To achieve this, all activities were designed in a playful, game-like format, mostly performed together in groups and adapted to the camp’s overarching theme, circus. A jumping castle, an air track, and other playful materials were available throughout the intervention for various games. Each day had its own sub-theme (e.g., acrobat) where children could earn stickers by participating in the activities linked to that theme. Breaks were relaxing, with access to games, books, and music. Once during each intervention children shared a treat of fries or ice cream together. |
| Role of parents | The intervention targeted the children, there was no separate parent module or education. However, before agreeing to enroll their child in the intervention, parents were informed about the aim and approach of the intervention. Furthermore, during the intervention, parents were frequently present and closely involved in interactions with the individual therapists when dropping off and picking up their children. They also had the opportunity to interact with other parents. On the final day of the intervention, parents could attend a performance where their children could demonstrate the activities they had learned during the intervention. |
| Camp principle | Children attended the camp daily, with the option of before- and after-camp care, but without standard overnight stay. If an overnight stay was explicitly requested, options were explored. However, none of the participants stayed overnight. An example of a day schedule is provided in Table 1. |
