# Supplementary material for: Parental perspectives on the changes in their child’s participation in physical activities after a highly intensive functional balance training for Developmental coordination disorder: A sequential multimethod qualitative study
Source: PLoS One. 2026 May 14;21(5):e0331994. doi: 10.1371/journal.pone.0331994 (PMC13175460; doi:10.1371/journal.pone.0331994)
Supplement: S2 File — (DOCX) [file pone.0331994.s002.docx]

**S3_File: Details on recruitment, selection criteria and descriptive characteristics of the child sample**

Recruitment of participants

Children were recruited through paediatric physical therapists across Flanders (Belgium), the Flemish non-profit organization for parents of children with DCD (V.Z.W. Dyspraxis), flyers, social media, and the study website. Spontaneous and out-of-region applications were also considered.

Selection criteria:

**Inclusion criteria:** Children were included if they were aged between 6 and 12 years old at start of the intervention and had a formal diagnosis of DCD or probable DCD based on the diagnostic criteria described in the Diagnostic and Statistical Manual of Mental Disorders, Fifth edition (DSM-5)(1): (i) motor skill acquisition and performance is lower when compared their age-matched peers (Criterion A), objectified with the total Movement Assessment Battery for Children, second edition (MABC-2) score at or below the 16th percentile or subscale score at or below the 5th percentile; (ii) the motor skills deficit significantly and persistently interferes with the activities of everyday living (Criterion B), objectified with the DCD Questionnaire 2007 (DCD-Q); (iii) Onset of symptoms in early childhood (Criterion C), evaluated by parental anamnesis; (iv) The motor skills deficits are not better explained by another medical (neurological, intellectual, visual, etc.), neurodevelopmental, psychological, or social condition, or cultural background (Criterion D), evaluated by a neuromotor examination performed by an acknowledged paediatrician.

Children who had no formal diagnosis or were on a waiting list for a diagnosis were evaluated by the research team using the diagnostic criteria as outlined in the inclusion criteria. In this case, Criterion D was evaluated based on anamnesis and clinical examination. If criterion A, B, C were met and no intellectual, visual or neurological conditions were reported, they were included as having “probable” DCD (2, 3).

Due to the intervention’s focus on postural control, only children scoring below the 50th percentile on the balance subscale of the Movement Assessment Battery for children, 2nd edition (MABC-2) were included. The presence of co-occurrences was no reason for exclusion, these were listed via anamnesis.

**Exclusion criteria:** Children are excluded if they are not able to follow instructions or cooperate sufficiently due to behavioural problems or when they do not meet all the aforementioned criteria.

Child sample of Phase 1 and Phase 2

|  | **Phase 1 (N=23)** | **Phase 2 (N=12)** |
| --- | --- | --- |
| Median age (years) | 7.9 | 7.9 |
| Sex (F:M) | 5:18 | 2:10 |
| Co-occurrences (yes:no) | 15:8 | 4:8 |
| ADHD (n) | 6 | 2 |
| Autism (n) | 6 | 0 |
| Autism + ADHD (n) | 3 | 2 |

N: total sample, n: subsample, F: Female; M: Male; ADHD: attentional deficit and hyperactivity disorder; Autism: Autism Spectrum Disorder

1. APA APA. Diagnostic and Statistical Manual of Mental disorders, 5th edition.: American Psychiatric Association; 2013.

2. Blank R, Barnett AL, Cairney J, Green D, Kirby A, Polatajko H, et al. International clinical practice recommendations on the definition, diagnosis, assessment, intervention, and psychosocial aspects of developmental coordination disorder. Developmental Medicine and Child Neurology. 2019;61(3):242–85.

3. Velghe S, Rameckers E, Meyns P, Johnson C, Hallemans A, Verbecque E, et al. Effects of a highly intensive balance therapy camp in children with developmental coordination disorder - An intervention protocol. Res Dev Disabil. 2024;147:104694.
