# Supplementary material for: Parental perspectives on the changes in their child’s participation in physical activities after a highly intensive functional balance training for Developmental coordination disorder: A sequential multimethod qualitative study
Source: PLoS One. 2026 May 14;21(5):e0331994. doi: 10.1371/journal.pone.0331994 (PMC13175460; doi:10.1371/journal.pone.0331994)
Supplement: S3 File — (DOCX) [file pone.0331994.s003.docx]

**S4_File: Open-ended questionnaire pre- (A) and post-interventional (B)**

### A. Pre-interventional questionnaire

Dear parent,

Through this survey, we aim to understand your experiences and perspectives regarding your child. All questions are open-ended. Please answer them as thoroughly as possible and include concrete examples where possible.

Thank you very much for your response!

- Parent’s name:
- Child’s name:

**Child**
This section explores your view on how movement difficulties affect your child.

1. How does your child perceive their own motor skills? Does this match their actual performance? Please explain with one or more examples.
2. How would you describe your child’s desire to move? Please explain with one or more examples.
3. To what extent does your child enjoy physical activities? Please explain with one or more examples.
4. How motivated is your child to try unfamiliar or less familiar physical activities? Please explain with one or more examples.
5. How would you describe your child's performance in activities like walking, running, jumping, climbing stairs, sports, etc.? Please explain with one or more examples.
6. How would you describe the interaction between your child and others during physical activities such as free play, sports, school, etc.? Please explain with examples.
7. Have there been any changes in your child’s activity pattern over the past six months? (e.g., started/stopped a new sport or therapy?)
8. Are there any other observations regarding your child's motor skills or self-image?

**Parent**
This section explores the impact of your child’s movement difficulties on you as a parent. The focus is on:

- *Practical matters:* e.g., changes in work schedule, extra daily support, etc.
- *Worries:* e.g., concerns about the future, school-related worries, etc.

1. What is the practical impact of your child’s movement difficulties on you as a parent? Please explain with examples.
2. What is the emotional impact of your child’s movement difficulties on you as a parent? Please explain with examples.
3. Are there other aspects of the impact on you as a parent you would like to elaborate on?

**Family**
This section addresses the impact of your child’s movement difficulties on your family. The focus is on:

- *Practical matters:* e.g., more time needed, extra care for siblings, different types of outings, etc.
- *Worries:* e.g., interaction with siblings, concerns about each other, etc.

1. What is the practical impact of your child’s movement difficulties on your family? Please explain with examples.
2. What is the emotional impact of your child’s movement difficulties on your family? Please explain with examples.
3. Are there any other family-related matters you’d like to elaborate on?

**Social interactions**

1. How does your child interact socially with peers? (e.g., during play, invitations to parties, etc.)

### B. Post-interventional questionnaire

Dear parent,

Through this survey, we aim to gain insight into your experience with your child and the effects of the camp. All questions are open-ended. Please answer them thoroughly and include concrete examples.
Thank you very much for your response!

- Parent's name
- Child's name

**Child**
This section explores the effects of the camp on your child's movement difficulties and the impact on your child.

1. Have you noticed any changes in how your child assesses their own motor skills? How does this compare to actual performance? Please explain with examples.
2. Have you noticed a change in your child’s desire to move? Please explain with examples.
3. Have you noticed a change in the joy your child experiences during movement? Please explain with examples.
4. Have you noticed a change in your child’s motivation to try unfamiliar/less familiar physical activities? Please explain with examples.
5. Have you noticed a change in your child’s performance in activities such as walking, running, jumping, climbing stairs, sports, etc.? Please explain with examples.
6. Have you noticed a change in your child’s interaction with others during physical activities, such as free play, sports, school, etc.? Please explain with examples.
7. Have you noticed other changes regarding your child’s motor skills or self-image?

**Parent**
This section explores the effect of the camp on how your child’s movement difficulties affect you as a parent. The focus is on:

- *Practical matters:* e.g., changes in work schedule, extra daily support, etc.
- *Worries:* e.g., concerns about the future, school-related worries, etc.

1. Have you noticed a change in the practical impact of your child’s movement difficulties on you as a parent? Please explain with examples.
2. Have you noticed a change in the emotional impact of your child’s movement difficulties on you as a parent? Please explain with examples.
3. Are there other changes you’ve noticed in how the movement difficulties impact you as a parent that you’d like to elaborate on?

**Family**
This section explores the effect of the camp on how your child’s movement difficulties affect the family. The focus is on:

- *Practical matters:* e.g., more time needed, extra care for siblings, different types of outings, etc.
- *Worries:* e.g., interaction with siblings, concerns about each other, etc.

1. Have you noticed a change in the practical impact of your child’s movement difficulties on the family? Please explain with examples.
2. Have you noticed a change in the emotional impact of your child’s movement difficulties on the family? Please explain with examples.
3. Are there other changes related to your family that you’d like to elaborate on?

**Social interaction**

Have you noticed a change in how your child interacts with peers? (e.g., during play, party invitations, etc.)
