# Supplementary material for: Parental perspectives on the changes in their child’s participation in physical activities after a highly intensive functional balance training for Developmental coordination disorder: A sequential multimethod qualitative study
Source: PLoS One. 2026 May 14;21(5):e0331994. doi: 10.1371/journal.pone.0331994 (PMC13175460; doi:10.1371/journal.pone.0331994)
Supplement: S4 File — (DOCX) [file pone.0331994.s004.docx]

**S5_File: Interview guide used during focus groups**

**Interview/Topic Guide**

Total time: 90 minutes

- 1. Introduction (5 min)
  2. Focus group (85 min)
- Opening Question (10 min)

Your children all participated in the camp this past August. Let’s begin by briefly introducing yourself and your child. How did your child experience the camp, and how did you experience your child's participation as a parent?

- Transition Question (10 min)

You were informed in advance about the topic of the focus group and asked to think about changes you observed. We will now create word clouds using Wooclap for each topic. First, we will generate the word clouds anonymously, and afterward, we’ll review the results together.

Please list the three biggest changes you noticed for each topic. If you didn’t notice any changes, you can enter "none.":

- CHILD: Name three changes you observed in your child.
- PARENT: Name three changes you observed in yourself as a parent.
- FAMILY: Name three changes you observed in your family (e.g., interactions, siblings...).
- SOCIAL INTERACTION: Name three changes you observed in your child’s social interactions.
- Main Questions (55 min)

(Question 1 & 2: 40–45 min; Question 3: 10–15 min)

The formulated main questions are from the researchers. Due to the organic flow of the conversation, Questions 1 and 2 will likely be discussed together. Some follow-up questions are provided under each main question to help guide the discussion during the focus group.

- **QUESTION 1** (+ question 2 = 45 min)

Various changes are observed within the child. In what ways do you think these are connected?

Let’s look at the word cloud for changes within the child. Are there things you recognize or don’t recognize?

Can you give an example of how you noticed these changes?

Was there a "magical" change that seemed to trigger other changes? Can you give an example?

Does the connection feel more like a loop/cycle or a domino effect?

Ask about changes not mentioned by parents:

“We know from earlier research using questionnaires that things like [findings questionnaires] could also increase or decrease after participating in the camp. Is this something you recognize?”

“How does this relate to the other changes?”

- **QUESTION 2** (+ question 1 = 45 min)

Do you see a connection between the changes in your child and those in yourself as a parent, your family, or your child’s social interactions?

Let’s look at the word clouds for parents, family, and social interactions. Are there things you recognize or don’t recognize?

Can you give an example of how you noticed these changes?

Do you feel these are connected to the changes within your child? How so?

Was there one or more specific changes within your child that triggered changes in you or your family? Can you give an example?

Does it also work the other way around? Did something change in you or your family that affected your child? Can you give an example?

- **QUESTION 3** (10–15 min)

Which elements of the intervention contributed to the observed changes?

(e.g., group setting, 1-on-1 coaching, high-intensity approach, being with other children with DCD, working on individual goals, location, circus theme…)

The camp differs in many ways from other camps. What do you think was the key to its success (or lack thereof) for your child?

We’re exploring whether the camps can be offered outside the research context. If you had to choose two components from the last camp to definitely include in future camps to help other children with DCD, which would you choose and why?

- Wrap-Up (10 min)

Observer visually shares key notes. The following questions are asked:

- Are there any other things you’d like to add?

What was the most important thing we discussed today (whether or not it’s already on the slide
