# Supplementary material for: Parental perspectives on the changes in their child’s participation in physical activities after a highly intensive functional balance training for Developmental coordination disorder: A sequential multimethod qualitative study
Source: PLoS One. 2026 May 14;21(5):e0331994. doi: 10.1371/journal.pone.0331994 (PMC13175460; doi:10.1371/journal.pone.0331994)
Supplement: S1 Table — (DOCX) [file pone.0331994.s005.docx]

| **Trustworthiness components** | **Strategy** | **Data collection** | **Data-analysis** | **Comment** |
| --- | --- | --- | --- | --- |
| **Credibility** |  |  |  |  |
|  | Prolonged engagement | ✓ | - | Data collection: in both phases, parents had been involved in the study for at least six months maintaining close contacts with the first authors through multiple quantitative assessments prior to qualitative data collection |
|  | Reflexivity | ✓ | ✓ | Data collection: personal biases were acknowledged among researchers during both phases. The design of the questionnaires and interview guides involved multiple researchers with efforts made to recognize and account for individual biases throughout the process.  Data-analysis: performed by at least two independent researchers with regular feedback from other members of the research team |
|  | Triangulation | ✓ | ✓ | Data collection: combination of open-ended questionnaires and focus groups  Data-analysis: conducted by at least two researchers, with consensus meetings involving a third researcher |
| **Transferability** |  |  |  |  |
|  | Thick descriptions | ✓ | - | Research context and methods are extensively described in the Method section, sample characteristics and context are provided in Table 1 and 2. |
|  | Sampling strategies | ✓ | - | Described in the Method section. |
| **Dependability** |  |  |  |  |
|  | Methodological documentation | ✓ | ✓ | Data collection and analysis: methodology is extensively described in the Methods section. |
|  | Audit trails | ✓ | ✓ | Data collection: each meeting and its decisions were documented in a written report  Data-analysis: each step of the analysis process was thoroughly documented by memos with reviewers’ thoughts and completion dates. |
| **Confirmability** |  |  |  |  |
|  | Peer debriefing | ✓ | ✓ | Data collection: multiple researchers were involved before and throughout data-collection to minimize personal biases  Data-analysis: performed by at least two independent researchers and a consensus meeting involving a third reviewer in both phases. Regular feedback from other members of the research team that were not directly involved in analysis, to help validate interpretations and introducing alternative perspectives |
|  | Member checking | ✓/🗶 | 🗶 | Data collection: in Phase 1, questionnaires findings were not shared with the participants. In Phase 2, participants received a brief summary of the discussion, allowing them to provide feedback or add insights. Full transcripts were not provided.  Data-analysis: Participants did not receive details of the data analysis process but will be provided with the complete study upon its completion. |
|  | Reflexive journaling | 🗶 | 🗶 | Data collection and analysis: although no formal reflexive journal was kept, the process was documented through memos including reviewers’ thoughts and completion dates |
|  |  |  |  |  |

**S1_ Table. Overview of trustworthiness components and applied strategies across data collection and analysis**
