# Supplementary material for: Parental perspectives on the changes in their child’s participation in physical activities after a highly intensive functional balance training for Developmental coordination disorder: A sequential multimethod qualitative study
Source: PLoS One. 2026 May 14;21(5):e0331994. doi: 10.1371/journal.pone.0331994 (PMC13175460; doi:10.1371/journal.pone.0331994)
Supplement: S2 Table — (DOCX) [file pone.0331994.s006.docx]

| **PHASE 1** | | | **BETWEEN PHASES** | **PHASE 2** | | | | |
| --- | --- | --- | --- | --- | --- | --- | --- | --- |
| Questionnaires | | Related Themes and Subthemes identified |  | Interview guide | | | | Related Themes and Subthemes identified |
| Domain | Topics questions |  |  | Transitioning | Main questions | | |  |
| Impact intervention on the **child:** | Motor behaviour (self-assessment motor skills, desire to move, motor performance)? | Theme 1: Child’s personal growth   - Self-esteem - Joy and interest in movement - Independence - Motivation - Estimate of own ability - Parental and child pride   Theme 2: Open up to new activities and making transfer   - Gross motor tasks - Performance of activities of daily living - Cycling - Individual goals - Activities in home context - Activities in school context - Activities in organized activities - Interaction with peers   Theme 3: Family understanding, knowledge and concerns   - Parental and child pride - Parental insights in coping with DCD - Insights of siblings in coping with DCD - Interaction siblings - Family dynamics - Parental concerns | Limitations Phase 1: some responses were brief, superficial or lacked contextual detail  Goals Phase 2:  - More detail into the findings of Phase 1  - Unravel how changes identified in Phase 1 interrelate | Name up to three changes you observed in your child. | Question 1: Various changes are observed within the child. In what ways do you think these are connected? | Question 2: Do you see a connection between the changes in your child and those in yourself as a parent, your family, or your child’s social interactions? | Question 3:  Which elements of the intervention contributed to the observed changes? | More detail in findings Phase 1:  - More acceptance of DCD diagnosis  - Research context  - Increased understanding and knowledge  How do perceive changes interrelate?  Theme 1: Child empowerment   - Interpreting - Experiencing - Self-regulation   Theme 2: Increased participation involvement through self-initiated actions   - Influencing - Responding - Choosing - Engaging - Acting   Theme 3: Parental centered support:   - Providing - Regulating - Informing   Theme 4: Empowerment induced sustained changes after three months  - Onset |
|  | Socio-emotional factors (joy during movement, motivation, self-esteem)? |  |  |  |  |  |  |  |
|  | Social interaction in physical activities |  |  |  |  |  |  |  |
|  | Other? |  |  |  |  |  |  |  |
| Impact intervention on the **parent:** | Effect on change in practical impact of movement difficulties? |  |  | Name up to three changes you observed in yourself as a parent. |  |  |  |  |
|  | Effect on change in emotional impact of movement difficulties? |  |  |  |  |  |  |  |
|  | Other changes on the parent? |  |  |  |  |  |  |  |
| Impact intervention on the **family:** | Effect on change in practical impact of movement difficulties? |  |  | Name up to three changes you observed in your family |  |  |  |  |
|  | Effect on change in emotional impact of movement difficulties? |  |  |  |  |  |  |  |
|  | Other changes on the family? |  |  |  |  |  |  |  |
| Impact intervention on **social interaction** of the child? | Changes in broader social interaction (motor and non-motor activities)? |  |  | Name up to three changes you observed in your child’s social interactions. |  |  |  |  |
