# Supplementary material for: Parental perspectives on the changes in their child’s participation in physical activities after a highly intensive functional balance training for Developmental coordination disorder: A sequential multimethod qualitative study
Source: PLoS One. 2026 May 14;21(5):e0331994. doi: 10.1371/journal.pone.0331994 (PMC13175460; doi:10.1371/journal.pone.0331994)
Supplement: S3 Table — (DOCX) [file pone.0331994.s007.docx]

**S7_Table: Definitions of processes among constructs described by the family of participation related constructs(1)**

| **PERSON-FOCUSED PROCESSES** | **Between constructs** | **Definitions as described in Imms et al. 2017 (1)** |
| --- | --- | --- |
| **Within-intrinsic factors** |  |  |
| Interpreting | Sense of self - preferences | Interpretation of past/current experiences in relation to the sense of self and competence influences development of preferences |
| Experiencing | Sense of self – activity competences | Individual experiences a sense of competence (or not) which colours his/her sense of self |
| Self-regulation | Overarching preferences, sense of self and activity competence | Binds together the intrinsic factors. Processess that enable the individuel to direct and monitor their, thinking, emotions, actions and interactions in the perceived context |
| **Participation vs. intrinsic factors** |  |  |
| Acting | Participation – activity competence | Children are more likely to participate in activities in which task competencies have been learned, spontaneously |
| Learning | Participation – activity competence | Participation might be encouraged by the assistance of skilled peers or adults (parents/teachers/therapists) |
| Perceiving | Participation – sense of self | Sense of self evolves as a result of participation, involves imagining one’s ability or opportunity to participate |
| Engaging | Participation – sense of self | Perceptions of self can predict future participation (internal state) |
| Choosing | Participation – preferences | Children choose what they will participate in (e.g. a preferred sport), actively choosing to participate |
| Complying | Participation – preferences | Cope with choices made by others (e.g reading at school) |
| **ENVIRONMENT-FOCUSED PROCESSES** | **Between constructs** | **Definitions*** |
| Providing | Environment – participation context | Environment’s actions to provide new participation contexts |
| Regulating | Environment – participation context | Environment’s actions within already existing participation context |
| Responding | Child – participation context | Child’s reactions to already existing participation contexts |
| Influencing | Child – participation context | Child’s self-initiate actions within participation context or to start new participation contexts |

1. Imms C, Granlund M, Wilson PH, Steenbergen B, Rosenbaum PL, Gordon AM. Participation, both a means and an end: a conceptual analysis of processes and outcomes in childhood disability. Dev Med Child Neurol. 2017;59(1):16–25.
